# Caregiving Across the Life Course: Life History Findings from the Health and Retirement Study (HRS)

**DOI:** 10.1093/geroni/igab046.2919

**Published:** 2021-12-17

**Authors:** Marina Larkina, Jacqui Smith

**Affiliations:** 1 University of Michigan, Ann Arbor, Michigan, United States; 2 University of Michigan, University of Michigan, Michigan, United States

## Abstract

Informal caregiving, defined as unpaid care provided to a relative or friend with some sort of special need, is a topic of research across different disciplines. Previous research highlights the prevalence and heterogeneity of caregivers in terms of their age, gender, relationship with the care recipient, and the duration of care provision. However, most research focuses on a specific episode of caregiving. Little is known about the people who provide care to multiple recipients throughout their own life. To fill this gap, we examined data from the HRS Spring 2017 Life History Mail Survey (N = 3520; age range 50-101 yrs). Participants reported their relationship with people to whom they had provided unpaid care for ≥ 6 months (max 5) and listed the start and end years of care. Compared with people who had not provided care, caregivers (N = 1000, 28%) were more likely to be women, white, and currently widowed. They cared for their parents (67%), spouses (22%), children (11%), or other relatives (16%) and 30% reported providing care two or more times (M = 1.44, SD = 0.81). Respondents, who reported multiple episodes of caregiving were more likely to be women, widowed, aged between 25 and 50 at the time of first providing care. People who first cared for their spouse were less likely to report multiple caregiving episodes comparing with those who cared for parents or children. Future research will examine the health and well-being consequences associated with caregivers’ histories of providing unpaid care to others.

